# Supplementary material for: Comparative multiomics analysis of cell physiological state after culture in a basket bioreactor
Source: Sci Rep. 2022 Nov 23;12:20161. doi: 10.1038/s41598-022-24687-4 (PMC9686226; doi:10.1038/s41598-022-24687-4)
Supplement: Supplementary file 1 — Supplementary Information 1. [file 41598_2022_24687_MOESM1_ESM.zip › raw data/Transcriptomic raw data/7.AS/2.dasplot/TE1vsTC1/A3SS/12_ARRDC3_NW_023666049.1_34353934_34354088_-@NW_023666049.1_34353934_34354126_-@NW_023666049.1_34354436_34354598_-.pdf]

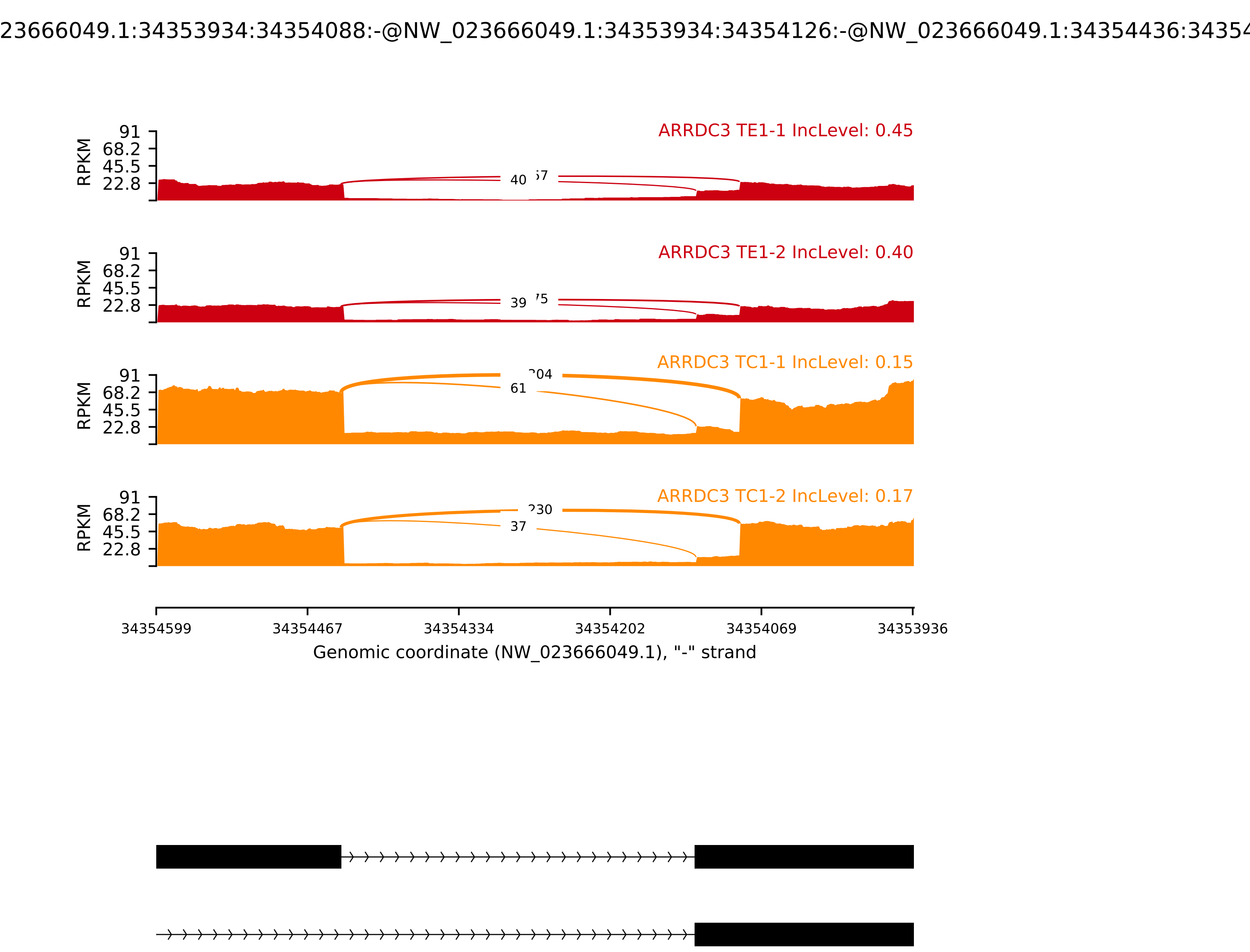
23666049.1:34353934:34354088:-@NW_023666049.1:34353934:34354126:-@NW_023666049.1:34354436:34354
ARRDC3 TE1-1 IncLevel: 0.45
ARRDC3 TE1-2 IncLevel: 0.40
ARRDC3 TC1-1 IncLevel: 0.15
ARRDC3 TC1-2 IncLevel: 0.17
RPKM
91
68.2
45.5
22.8
57
40
75
39
204
61
230
37
34354599
34354467
34354334
34354202
34354069
34353936
Genomic coordinate (NW_023666049.1), "-" strand
